# Monitoring of the copper persistence on plant leaves using pulsed thermography

**DOI:** 10.1007/s10661-022-09807-x

**Published:** 2022-02-08

**Authors:** Massimo Rippa, Valerio Battaglia, Michele Cermola, Mariarosaria Sicignano, Ernesto Lahoz, Pasquale Mormile

**Affiliations:** 1 Institute of Applied Sciences and Intelligent Systems “E. Caianiello” of CNR, via Campi Flegrei, 34, Pozzuoli (Na), 80072 Italy; 2 CREA - Research Centre for Cereal and Industrial Crops, via Torrino, 2, Caserta, 81100 Italy

**Keywords:** Fungicide, Tobacco, Grapevine, Precision Agriculture, Adjuvant

## Abstract

Copper-based fungicides are largely used in agriculture in the control of a wide range of plant diseases. Applied on plants, they remain deposited on leaf surfaces and are not absorbed into plant tissues. Because of accumulation problems and their ecotoxicological profiles in the soil, their use needs to be monitored and controlled, also by using modern technologies to better optimize the efficacy rendering minimum the amount of copper per season used. In this work, we test a novel approach based on pulsed thermography to evaluate the persistence of the copper on plant leaves so that the time between two applications should be the minimum needs. We monitored the thermal response observed on different treatments of both grapevine and tobacco plants over a 3-week period. Our experimental results demonstrate that the new methodological approach based on pulsed thermography can be an effective tool to evaluate in real time the presence of copper on differently treated plants allowing a tentative quantification and, therefore, to optimize its use in the agricultural practices, according also to the European Regulation n. 1107/2009.

**Supplementary information:**

The online version contains supplementary material available at 10.1007/s10661-022-09807-x.

## Introduction

Copper has been used in agriculture as a fungicide and bactericide for over a century. It plays a key role in integrated pest management, and it is widely used in organic farming (La Torre et al., 2018; Rusjan, 2012). Applied as a protective spray on plants, copper-based fungicide (CBF) remains deposited on leaf surfaces and is not absorbed into plant tissues. Its active ingredient, the cupric ion (Cu^++^), is soluble in water and provides antifungal and antibacterial effects at low concentration levels (Scortichini et al., 2018). However, due to the mechanical actions of wind, rain, or irrigation, the metallic material reaches the soil remaining in it as a contaminant for long periods causing bioaccumulation and toxicity to both microbial biomass and biodiversity in soil (Banu et al., 2004; Lamichhane et al., 2018; Komárek et al., 2010; Mackie et al., 2012). For this reason, the European Union by means of EU Regulation n° 1981 of December 13, 2018, has established maximum limits on CBF in organic farming at 28 kg ha^−1^ in 7 years. Different strategies to reduce and to optimize the use of CBF have been studied and tested at both industrial and research levels, like the use of innovative formulation based on reduced particle size of the active substance to improve coverage of treated surfaces (Brunelli & Palla, 2005), the use of CBF microencapsulates for controlled release (Weihrauch & Schwarz, 2014), the use of net crop covers to reduce the amounts of agrochemicals in crop protection (Scarascia et al., 2012; Alaphilippe et al., 2016), and the use of adjuvants to increase the cooper persistence on leaf (Flori et al., 2006; Orbovic et al., 2007). Despite satisfying results from an agronomical point of view, considering the EU regulation, new approaches and techniques to optimize the use of CBF are required. The growing interest in farming management based on the precision agriculture concept stimulates more and more the development and use of new techniques and new biological adjuvants, such as our polysaccharide-based adjuvant derived from locust bean gums (Lahoz et al., 2017) with the goal of optimizing returns on inputs while preserving resources. Organic farmers have little means to combat effectively downy mildew *Plasmopara viticola* (Berk. & M.A. Curtis) Berl. & De Toni except for CBF sprays (Finckh et al., 2015). Copper is the subject of new interest for two opposite reasons: the first is the increased interest for the use of natural compounds and organic farming; the second is the concern about its eco-toxicological profile and accumulation in the soil. This new scenario modified the phytoiatric use of CBF about rates and strategies, creating the need to reduce the total amount used. The number and interval of CBF applications depend on plant phenological stage, washout, risk of infection, and quality of distribution on leaves. Formulation and adjuvants could play an important role to increase bioavailability and regulate the frequency of application on grapevine. The addition of an appropriate adjuvant with a foliar fungicide can significantly improve coverage, absorption, and efficacy, and can reduce the total amount applied in a season. Adjuvants are additives used for many purposes: to increase persistence on leaves (Steurbaut, 1993), to regulate absorption and spray retention (Hart et al., 1992), for rainfastness (Kudsk, 1991), for foliar washoff and runoff losses (Reddy & Locke, 1996), and for pesticide translocation (Maschhoff et al., 2000). Moreover, adjuvants can influence the efficacy of pesticides (Grayson et al., 1996a, 1996b; Percichv & Nickelson, 1982; Rowen, 1979), obtaining contemporary economic and environmental benefits (Kirkwood, 1993).

Active thermography (AT) is a well-known non-invasive and contact-less imaging technique that represents an outstanding innovation applied in many fields aerospace, engineering, medicine, and veterinary and recently it is gaining great interest in agriculture (Capraro et al., 2017; Grant et al., 2006; Guilioni et al., 2008; Ishimwe et al., 2014; Pineda et al., 2021; Vadivambal & Jayas, 2011). It has been used in different agricultural applications to evaluate the physical and physiological characteristics of plants such as transpiration rates, heat capacity of the leaves, local water content, water flow velocity, and response to UV interaction (Garbea et al., 2002; Bajons et al., 2005; Blonquist et al., 2009; Bonanomi et al., 2017; Rippa et al., 2020). This technique fits well with the precision agriculture management strategy, and it represents a reliable means of providing a low-cost in situ analysis (Cohen et al., 2015; Lenthe et al., 2007; Meron et al., 2010; Oerke et al., 2011; Stoll et al., 2008). According to this technique, the surface of the sample under investigation is stimulated using an external heat source and its thermal response is detected and recorded using infrared camera.

In this paper, we propose a novel approach based on the pulsed thermography (PT) to monitor the persistence of CBF on leaf surfaces and the use of new adjuvants to prolong its persistence. The objectives of this work were (i) to use the PT to measure and to compare the thermal recovery times (TRt) of leaf surfaces of two different plant species, grapevine, and tobacco, treated with CBF; (ii) to monitor the variations of this parameter for 3 weeks in order to evaluate if this approach can be successfully used for the optimization of CBF treatments on the two species of plants under consideration; and (iii) to test the effect of a new sticker adjuvant on the persistence of CBF on plant leaves. To the best of our knowledge, this is the first time that a thermographic technique has been used for copper (or in general of a fungicide) persistence monitoring on leaves.

## Materials and methods

### PT measurements

**Fig. 1:**
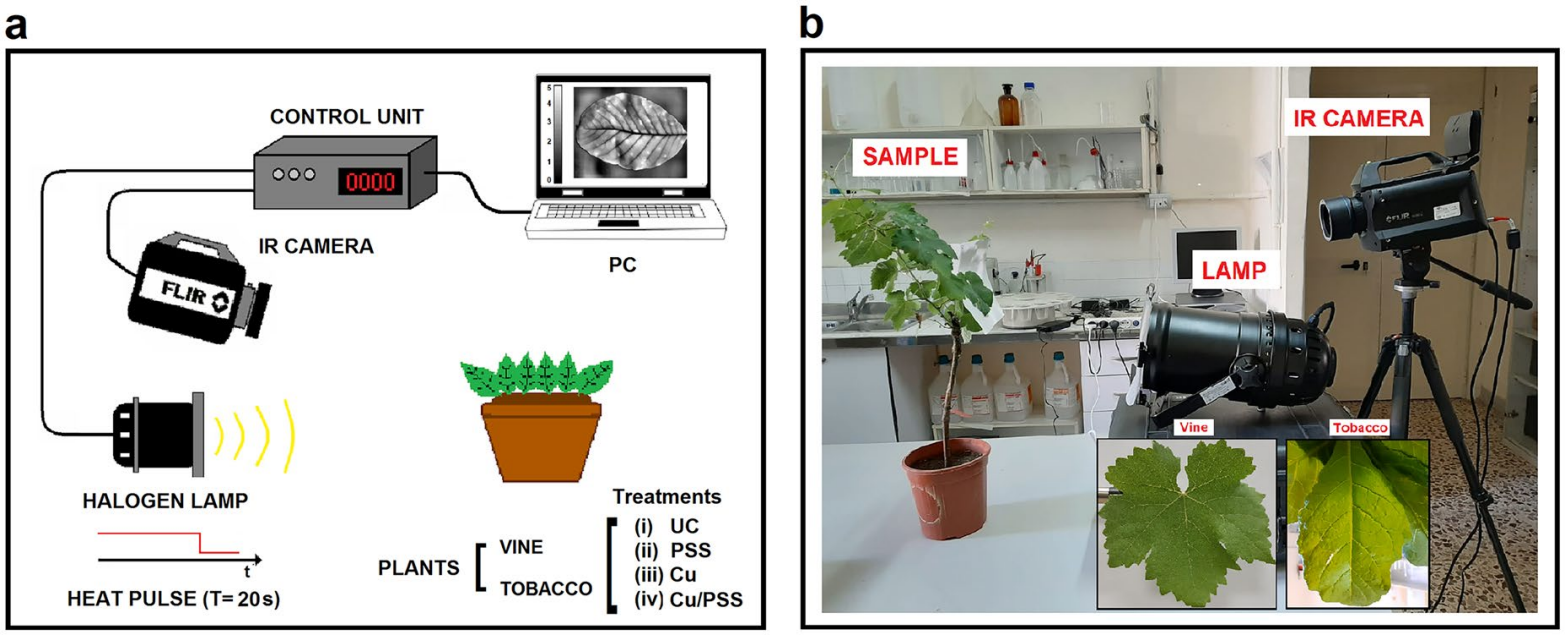
Experimental PT setup used: **a** scheme of the set-up, **b** photo of the setup, in inset the images of leaves belonging to the two plants species analyzed

PT measurements were performed using a halogen lamp with tunable power to generate a thermal pulse of 20 s on each leaf investigated. The illuminance on the leaves was measured and controlled using a photo-radiometer (Delta Ohm HD 2102.2) with a lux meter probe (LP-471-PHOT) to achieve an induced thermal gap lower than 10 °C. The thermal response achieved during and up to 120 s after heating was recorded with a frame rate of 5 Hz using a MWIR camera FLIR X6580 sc with a cooled indium antimonide (InSb) detector (spectral range 1.5–5.4 μm, FPA640 × 512 pixels and NETD ~ 20 mK at 25 °C) mounting a 50-mm focal lens with spectral band 3.5–5 μm and IFOV 0.3 mrad. Spatial maps of the TRt of the leaves were calculated using a home-made MATLAB code (R2019b, MathWorks) analyzing the temporal trend of the temperature from the frames acquired. The TRt associated with each pixel of the map is defined as the time that it spent to recover 80% of the induced thermal gap by the heating pulse. The 80% threshold represents the value that in our experimental measurements allows to achieve a higher resolution of the temporal data. The measurements were carried out under controlled environmental conditions with a room temperature of 25 °C and humidity of 55%. In Fig. 1a, b are shown respectively a scheme and a picture of the experimental setup used.

### Plants: material, treatment, and residue analysis

#### Plants investigated

Grapevine (*Vitis vinifera* L.) and tobacco (*Nicotiana sylvestris* L.) plants were used for the experiment. Grapevine seedlings of ‘Aglianico amaro’ (*V. vinifera* L.) variety were cultivated with two shoots for each plant, and each shoot had 8 leaves so that they developed 16 leaves. Each treatment consisted of 6 plants. Plants of ornamental tobacco (*N. sylvestris* L.) with 10 leaves were used for the experiment. Each treatment consisted of 6 plants. All grapevine and tobacco plants were grown in a greenhouse in 15-cm pots filled with a pasteurized mixture of soil (zeoliter50) and sand (1:1, v:v). The mixture was pasteurized at 75 °C for 1 h on two consecutive days by autoclaving. All plants were kept at 25 °C with a 12-h photoperiod and watered as required. Protocol and rates of applied products are also reported in Table 1.

**Table 1:** Protocol and rates of applied product on grapevine and tobacco plants

Treatments	Commercial product	Formulation	Field rate (g ha^−1^)	H_2_O per plant (mL)	Rate per plant of copper fungicides and /or PSS (g)
Sterile distilled water (UC)	-	-	-	200	-
Copper fungicide (CU)	Airone Extra (Gowan Italia)	Copper hydroxide (20%) + copper oxychloride (10%)	1.500	200	0.6
Copper fungicide + Locust bean gum (Cu/PSS)	Airone Extra (Gowan Italia) + PSS	Copper hydroxide (20%) + copper oxychloride (10%) + PSS	1.500 + 5.000	200	0.6 + 2
Locust bean gum (PSS)	PSS	PSS	5.000	200	2

#### Treatments compared

The trial includes (i) plants treated with sterile distilled water (UC); (ii) plants treated with CBF Airone extra (Gowan) containing 30% copper (Cu); (iii) plants treated with a natural adjuvant based on galactomannan extracted from locust bean gum plus CBF (Cu/PSS); and (iv) plants treated with a natural adjuvant from locust bean gum alone (PSS). Grapevine and tobacco plants were sprayed with the products using a glass atomizer and were kept in a greenhouse at 25 °C. The plants were sprayed once in order to test the persistence of CBF on leaves after a single application. The application of the products was done on grapevine when leaves of seedlings were completely developed (BBCH 15) and on tobacco when plants had 10 leaves opened (BBCH 1110).

#### Residue analysis

After 21 days from the application, residue analyses regarding fungicide and copper were performed on leaves by a private company (Laboratoria, Naples, Italy). The analysis was made according to references present in literature (Čuš et al., 2010; OIV, 2020).

## Results and discussion

In this study, we used the PT technique to monitor the persistence of CBF on treated plant leaves vs. time. We analyzed the leaves of two species of plants, grapevine, and tobacco, comparing the results achieved on (i) plants treated with sterile distilled water (UC) and plants treated in the following three ways: (ii) with CBF (Cu), (iii) with a natural adjuvant based on galactomannan extracted from locust bean gum plus CBF (Cu/PSS), and (iv) with the natural adjuvant from locust bean gum alone (PSS).

As recently demonstrate (Lahoz et al., 2017), the use of PSS increases the persistence of copper on the leaves. The basic idea that inspired our methodological approach, aimed to monitor the CBF persistence on the leaves, occurred from the basic physical principle that the thermal properties of each body depend on its chemical composition. Due to the presence of metal residues, after a CBF treatment on a plant, the leave surface changes its physical properties, and these variations affect the thermal response that can be detected by our acquisition system.

**Fig. 2:**
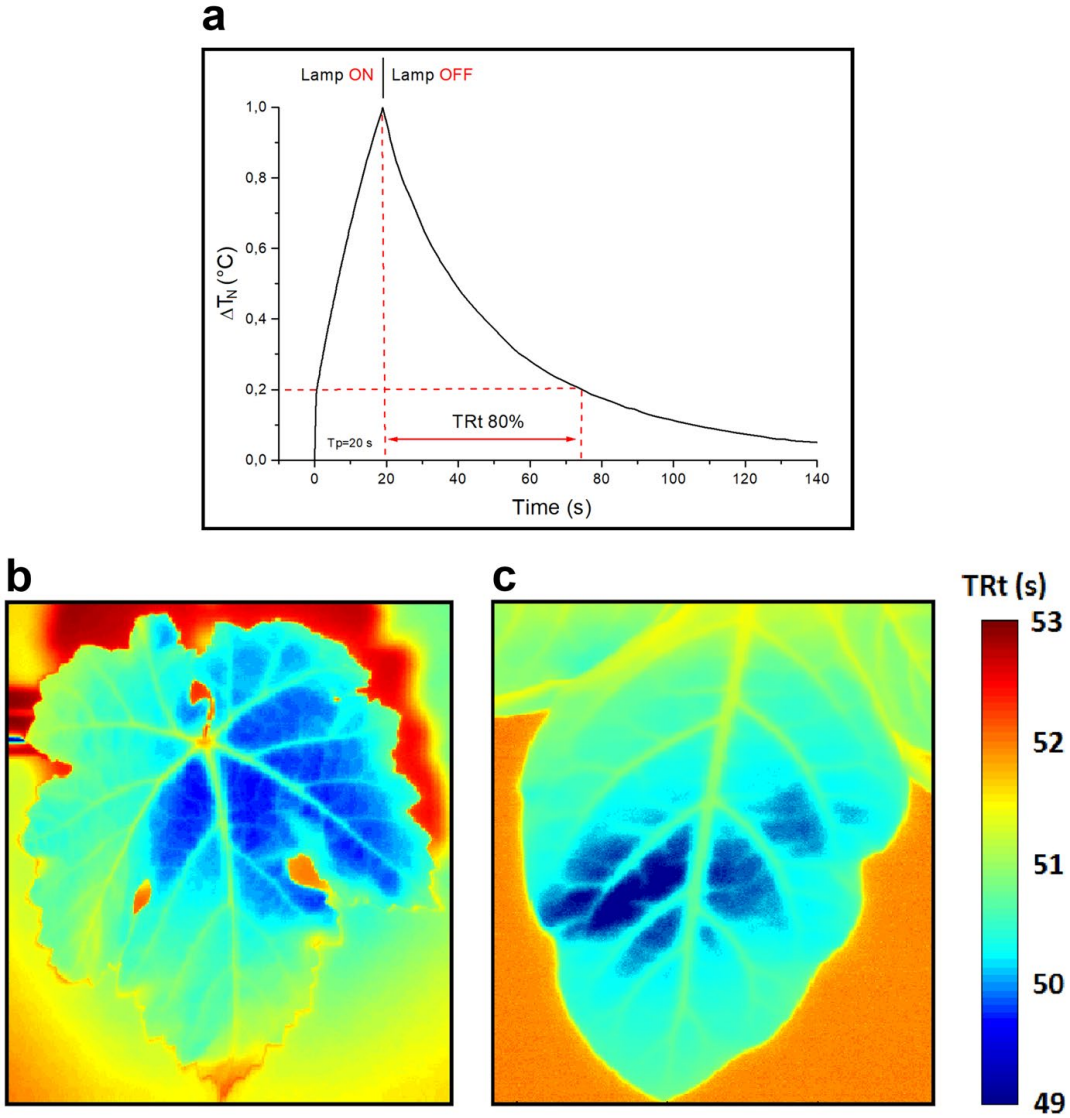
TRt analysis: **a** example of temporal trend of the normalized thermal gap recorded on a single pixel, in the graph with *T*_*p*_ is indicated the pulse period of 20 s. Spatial maps of the TRt calculated for two leaves with Cu treatment: **b** grapevine leaf and **c** tobacco leaf

In our experimental approach, leaves of the plants under investigation are heated using a controlled thermal pulse. Infrared images are recorded during and after this stimulation, and from the thermal frames acquired, we extrapolate the temporal trend of the temperature for each single image pixel. To better clarify our methodology, in Fig. 2a, we report an example of temporal graph relative to the normalized thermal gap (Δ*T*_*N*_) of a pixel. From this trend, we evaluate the TRt associated with the pixel as defined in “Materials and methods”. Calculating this parameter for each pixel, we can achieve a spatial map relative to the TRt distribution. In Fig. 2b, c, examples of spatial maps of the TRt relative to a grapevine and tobacco leaf both treated with CBF are reported.

In both maps, according to the different local thermal properties, the areas of the leaves that correspond to the lowest TRt values (azure-blue colors) are those mainly characterized by the presence of copper residues. In fact, the higher conductivity of areas with metal residues results in faster thermal responses and therefore lower recovery rates. We do observe that, as a preliminary analysis, in the present study we tested and compared different thermographic approaches to determine the most suitable one for monitoring the persistence of copper, which is the aim of this work. In addition to the approach based on PT, we evaluated also both the passive approach and another active thermography technique, the well-known lock-in thermography (LiT). We report and discuss the comparison of the results achieved with the thermographic techniques tested in the supplementary file. Despite interesting results obtained with the LiT, comparable with those shown in Fig. 2, for our study we preferred to focus on the PT technique which is certainly easier to implement, also in view of using the proposed method in field or for the realization of a prototype monitoring system. In fact, in the LiT technique there is the necessity to monitor the exact time dependence between the output signal and the modulated heating and this requires dedicated hardware to control and to set the lamps. Moreover, other technical parameters as the thermal wave period and the recording frame rate must be set accurately. These technical complications are reduced in the case of the PT technique that, when it provides results comparable to those of LiT, makes it preferable for its simple use.

**Fig. 3:**
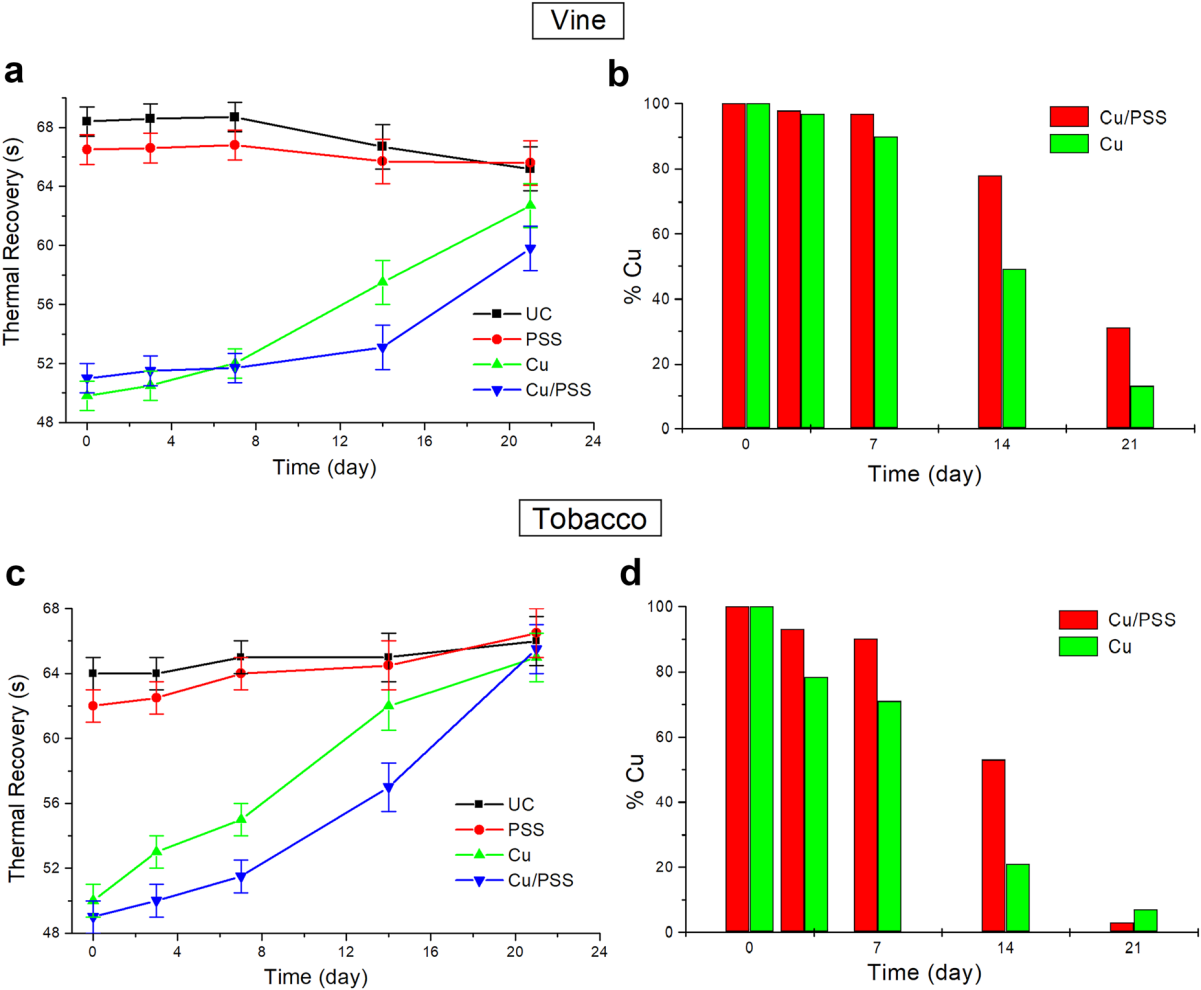
TRt analysis realized on UC (black squares), PSS (red circles), Cu (green up triangles), and Cu/PSS (blue down triangles) treatments for **a** grapevine plants and **c** tobacco plants. %Cu estimated during the monitoring for Cu and Cu/PSS treatments: **b** vine plants and **d** tobacco plants

For each plant (treated and untreated), we monitored three leaves obtaining TRt evaluations over a 3-week period. In Fig. 3, we report the results relative to the TRt values observed on the two plants species considered.

Graphs 3a and 3c show the TRt versus the days measured respectively for the grapevine and tobacco plants treated. In the graphs, each point represents the average TRt value calculated on the whole area of the three leaves monitored. Error bars were calculated as the standard deviation of the experimental evaluations realized. As shown in the graphs, for the two species of plants investigated, the trends that correspond to the same treated samples are quite similar. The UCs (black squares) show TRt values enough constant in the time. The low variability observable is mainly due to the increase in both area size and thickness of the leaves in the period of observations. An increase in the size of the leaf area produces a higher contribution of the convection to the heat transfer allowing a faster cooling of the leaf and therefore lower TRt. On the contrary, the increase of the thickness of the leaves, and therefore of their bulk mass, produces a higher inertia to the heat dissipation resulting in a slower cooling of the leaves and, hence, higher TRt values. The decrease of TRt values observed for the UC sample of grapevine (in Fig. 3a) and the increase of the same parameters for the tobacco sample (in Fig. 3c) are therefore explained by the prevalence of one of the factors described above.

The TRt measured for the plants treated only with PSS (red circles) shows trends and values like that found for the UC, because this gel is essentially water based (~ 90%) with a thickness on the leaf surface of 2–3 microns. These physical characteristics do not affect considerably the thermal properties of the leaves with respect to the UC case. In the case of plant Cu treated, with (blue down triangles) and without PSS (green up triangles), the TRt achieved at time *t*_0_ are respectively about 20 s for grapevine plants and about 15 s for tobacco plants lowers than those measured for the respective UCs. Successively, they increase monotonically over the 21 days of monitoring due to the reduction of the presence of CBF on the leaf surface, converging toward to those of the UC after 3 weeks of monitoring. Comparing the trends found for Cu and Cu/PSS, for both species of plants, the higher persistence of CBF in the presence of the PSS action entails, for the plants with the adjuvant, a slower increase in TRt values in the first 2 weeks of observations. To do a tentative quantification of the presence of copper on the leaves monitored, we calculated the percentage of the metal residue on the leaves using the following relation:

1 $$\%Cu\left(t\right)=\frac{{TRt}_{X}\left(t\right)-{TRt}_{UC}\left(t\right)}{{TRt}_{X}\left(0\right)-{TRt}_{UC}\left(0\right)}$$

where *TRtx*(*t*) and *TRtx*(*0*) are the TRt found for Cu or Cu/PSS treatments respectively at day *t* and at day 0, while *TRt*_*UC*_(*t*) and *TRt*_*UC*_(*0*) are the same quantities for UCs. In Fig. 3 b and d are reported the histograms relative to the %Cu estimated for both Cu (green bar) and Cu/PSS (red bar) treatments during the observation period for both grapevine and tobacco plants. The maximum difference in the %Cu between the plants Cu treated and Cu/PSS treated is observable after 2 weeks for both species, and it is respectively about 29% for grapevine plants and 32% for tobacco plants. These results demonstrate that the new polysaccharide-based adjuvant acting as a sticker has the ability to prolong the persistence on leaves of CBF used in this work.

In both Cu and Cu/PSS treatments, the level of copper residues registered 21 days after the application was less than 2.5 mg kg^−1^ and, hence, under the minimum residue level permitted by law. The latter achievement confirms that even if the PSS acts as a retarder of the CBF degradation, it does not affect the duration of preharvest interval.

We point out that the conventional methods for the determination of copper residues in plants involve analytical techniques based on atomic absorption spectrometry (AAS) (European Food Safety Authority, 2018). However, as well known, these methods are expensive, are time-consuming, require highly specialized staff, are destructive, and do not allow in-field analysis. Other alternative strategies for the copper residue detection in plants based on the use of imaging techniques have been proposed in literature in few exploratory studies (García-Martín et al., 2020; Goswami & Das, 2017; Mijovilovich et al., 2020; Zeng et al., 2019). However, compared to the imaging approach that we implement in this work, these methods require analyses both in spectral ranges and with operational protocols more complicated and expensive and which is difficult to implement a prototype monitoring system that works in field. We think that the method we propose based on the PT technique represents a valid alternative strategy, different from those conventionally used or present in the literature, and which opens up the possibility of an investigation of metal residues on plants in field and in real time. Although our results are limited to two plant species, they highlighted how the analysis strategy based on the use of the PT technique could be an effective tool to monitor the presence of CBF on differently treated leaves and, therefore, to optimize its use in agricultural practice.

## Conclusions

In this work, we used PT to monitor the presence of copper on leaves surface of grapevine and tobacco plants. We compared the TRt values measured on different plants treatments over a 3-week period. Monitoring this parameter, we controlled the presence of the CBF giving a quantification of the metal residue. Our achievements demonstrate how the PT technique can be used to monitor the persistence of the copper on plant leaves and to test the effectiveness of novel natural adjuvants. Tools of analysis based on this strategy can contribute to an optimized use of copper allowing both to reduce the number of treatments and to develop a more sustainable agriculture with higher environmental safeguard. At the same time, the approach we present here meets the needs of emerging research in precision agriculture, concerning the development of new techniques capable of providing information in real time. Moreover, as a further result of this work, we demonstrated the ability of the new gel adhesive to delay CBF degradation thus representing a possible useful means to reduce both the total seasonal quantity and the number of CBF applications normally used to control fungi diseases on the leaves.

## Supplementary Information

Below is the link to the electronic supplementary material.

Supplementary file1 (DOCX 162 KB)

## Data Availability

Data are available from the authors upon reasonable request.
