# Supplementary material for: Monitoring of the copper persistence on plant leaves using pulsed thermography
Source: Environ Monit Assess. 2022 Feb 8;194(3):160. doi: 10.1007/s10661-022-09807-x (PMC8825608; doi:10.1007/s10661-022-09807-x)
Supplement: Supplementary file 1 — Supplementary file1 (DOCX 162 KB) [file 10661_2022_9807_MOESM1_ESM.docx]

**Supplementary Informations**

Monitoring of the Copper persistence on Plant Leaves using Pulsed Thermography

Massimo Rippa^1^, Valerio Battaglia^2^, Michele Cermola^2^, Mariarosaria Sicignano^2^, Ernesto Lahoz^2^, Pasquale Mormile^1^

^1^ Institute of Applied Sciences and Intelligent Systems “E. Caianiello” of CNR, I-80072 Pozzuoli (Na), Italy

^2^ CREA - Cereals and Industrial Crops Research Centre, via Torrino, 2, I-81100 Caserta, Italy

**Preliminary thermographic tests**

We realized preliminary tests to compare different thermographic approaches in order to determine the most suitable one for monitoring the persistence of Cu, aim of our work. In addition to the approach based on pulsed thermography (PT), we evaluated both passive approaches and other active thermography techniques including the well-known Lock-in thermography (LiT). As an example, the figure S1 below shows and compares 4 images that we achieved of a tobacco leaf treated with copper based fungicides (CBF) that represent: a) the map of the thermal recovery times (TRt) obtained with the PT analysis (also shown in the manuscript), b) the passive thermal image achieved in natural environmental conditions, while c) and d) are respectively the phase and amplitude maps obtained with the LiT technique.


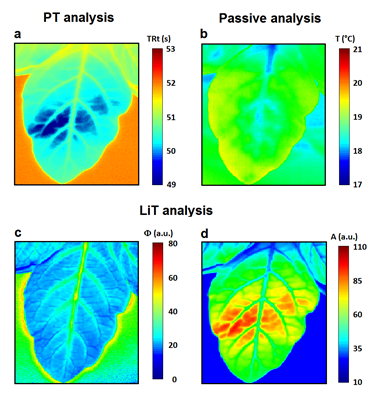


**Fig. S1:** Comparison of Termographic techniques to detect the presence of copper residue on a tobacco leaf: a) map of the thermal recovery times (TRt) obtained with the PT analysis, b) passive thermal image achieved in natural environmental conditions, c) phase and d) amplitude maps obtained with the LiT technique.

As described in the paper, in the case of figure S1a the areas of the leaves mainly characterized by the presence of Cu are well visible and characterized by lowest TRt values (azure-blu colors). This is due to the different optical and thermal properties of the metal residue (lower emissivity and higher conductivity) respect the leaf surface that produces a faster thermal response.

Differently, as clearly visible from the figure S1b, the thermal map of the leaf obtained with the passive approach does not show appreciable signals associable with the presence of Cu. In fact, as expected, under natural environmental conditions, the fungicide thermalizes with the leaf surface thus making it impossible to detect it with this approach.

In the case of LiT, the plants were heated using 3 cycles of a halogen lamp (200 W) square wave modulated with a period T = 40 s (25 10−3 Hz). During heating, thermal images were acquired with a frame rate of 5 Hz. From recorded data, phase and amplitude maps were calculated using the software IRTA (DES). The phase map in figure S1c gives a clear view of the morphological structure of the leaf but no information relating to the presence of Cu on the surface. As well known, the phase parameter is related to the propagation time of the thermal wave inside the sample under investigation and it is independent of local optical and infrared surface features (as for example the emissivity) [1-2]. Therefore, this intrinsic characteristic of the phase makes it impossible to detect the Cu, the presence of that mainly alters the thermo-optical phenomena concerning the leaf surface. Differently, the amplitude map in figure S1d is strictly dependent on the surface thermo-optical parameters (such as diffussivity and emissivity) and hence it allows to see the presence of the metal residue in a similar way as the TRt map (figure S1a).

We observe that despite this last interesting result obtained with the LiT, for our study we preferred to focus on the PT technique which is certainly easier to implement, also in view of using the proposed method in field or for the realization of a prototype monitoring system (next objective of our research). In fact, in the LiT technique there is the necessity to monitor the exact time dependence between the output signal and the modulated heating and this requires dedicated hardware to control and to set the lamps. Moreover, other technical parameters as the thermal wave period and the recording frame rate must be set accurately. These technical complications are very reduced in the case of the PT technique that, when it provides results comparable to those of LiT, makes it preferable for its simple use.

1. X.P.V. Maldague. Theory and Practice of Infrared Technology for Nondestructive Testing. Wiley-Interscience; 1st edition (April 30, 2001). ISBN: 978-0-471-18190-3.

2. C. Meola, G. Carlomagno. Recent advantages in the use of infrared thermography. Measurement, Science and Technology, pp. 27-58., 2004.
